# MicroRNA-31 functions as a tumor suppressor and increases sensitivity to mitomycin-C in urothelial bladder cancer by targeting integrin α5

**DOI:** 10.18632/oncotarget.8479

**Published:** 2016-03-30

**Authors:** Tianyuan Xu, Liang Qin, Zhaowei Zhu, Xianjin Wang, Yue Liu, Yong Fan, Shan Zhong, Xiaojing Wang, Xiaohua Zhang, Leilei Xia, Xiang Zhang, Chen Xu, Zhoujun Shen

**Affiliations:** ^1^ Department of Urology, Ruijin Hospital, School of Medicine, Shanghai Jiaotong University, Shanghai, China; ^2^ Department of Urology, The First Affiliated Hospital of Zhengzhou University, Zhengzhou, China; ^3^ Shanghai Key Laboratory of Reproductive Medicine, School of Medicine, Shanghai Jiaotong University, Shanghai, China

**Keywords:** microRNA-31, integrin α5, bladder cancer, chemotherapy, mitomycin-C

## Abstract

Urothelial bladder cancer (UBC) is a common genitourinary malignancy. MiR-31, a well-identified miRNA, exhibits diverse properties in different cancers. However, the specific functions and mechanisms of miR-31 in UBC have not been investigated. In this study, tumor samples, especially invasive UBC, showed significantly reduced level of miR-31, as compared with normal urothelium. Prognostic analysis using the EORTC model showed that down-regulation of miR-31 correlated with higher risks of recurrence and progression in noninvasive UBC cases. Remarkably, overexpression of miR-31 mimics in UBC cell lines inhibited cell proliferation, migration and invasion. Integrin α5 (ITGA5), an integrin family member, was subsequently identified as a direct target of miR-31 in UBC cells. When treated with mitomycin-C (MMC), miR-31-expressing UBC cells displayed lower survival and higher apoptotic rates, and deactivated Akt and ERK. These effects arising from miR-31 overexpression were abrogated by ITGA5 restoration. Furthermore, miR-31 markedly inhibited tumor growth and increased the effectiveness of MMC in UBC xenografts. In summary, our data suggest that miR-31 is a prognostic predictor and can serve as a potential therapeutic target of UBC.

## INTRODUCTION

Bladder cancer is the fourth most frequent male malignancy in the United States and the most common genitourinary tract cancer in China. There are approximate 429,800 new cases and 165,100 deaths annually worldwide [1]. More than 90% of bladder cancers are classified as urothelial bladder carcinomas (UBC), most of which present as the noninvasive form (stage Ta–T1). Noninvasive UBC is routinely treated with transurethral resection of bladder tumor (TURBT) and intravesical chemotherapy. However, more than 50% of tumors will recur and 10-20% progress to invasive disease (stage T2–T4) [2]. Patients with invasive UBC are suggested to receive radical cystectomy and systematic chemotherapy. Unfortunately, they still have a poor 5-year survival rate of less than 60% [3]. One major dilemma in UBC treatment is inferior sensitivity or resistance to chemotherapy, but few advances have been made in clinical practice. Strategies based on new molecular targets are urgently needed in UBC diagnosis and therapeutics.

MicroRNAs (miRNAs) are a conserved class of small noncoding RNAs that regulate 30% of human genes by mRNA degradation or repression [4]. They are often aberrantly expressed and function as oncogenes or tumor suppressors in various cancers, including UBC. For example, UBC tissues show reduced expressions of miR-99a, miR-100, miR-101 and miR-145, all of which target fibroblast growth factor receptor 3. In invasive UBC, miR-21 and miR-373 are up-regulated and serve as oncomiRs by inhibiting the apoptotic p53 pathway [5]. Our previous study also demonstrates that miR-145 promotes cell apoptosis and inhibits proliferation and migration by suppressing insulin-like growth factor 1 receptor directly [6]. Another miRNA, miR-31, is well recognized as a pleiotropic miRNA in different cancer types [7–13]. Wang et al. [14] found that the miR-31 expression in UBC was lower than that in normal urothelium. Wszolek et al. [15] identified the down-regulation of miR-31 in invasive UBC tissues. These works strongly suggest a role of miR-31 in control of UBC. However, the functional mechanisms have not been elucidated.

Integrins are a family of transmembrane receptors that mediate cell-cell interactions, attachment to extracellular matrix (ECM) and signal transduction. In mammalian cells, there are 24 distinct integrin heterodimers formed by the combination of 18 α- and 8 β-subunits. Integrin subunits or heterodimers can modulate various biological behaviors, such as cell adhesion, proliferation, apoptosis and motility, and they make a substantial contribution to cancer development or drug resistance [16, 17]. In UBC, we have previously revealed that integrin β1 (ITGB1) is responsible for resistance to the chemotherapeutic drug mitomycin-C (MMC) [18]. Some integrins are known to be regulated by miRNAs. For instance, miR-320a serves as a prognostic factor and inhibits metastasis by targeting integrin β3 in salivary adenoid cystic cancer [19]. In human hepatocellular carcinoma, miR-26a promotes anoikis by targeting integrin α5 (ITGA5) [20]. Hence, miRNA/integrin axis may play a critical role in cancer biology.

In this study, we evaluated the specific role of miR-31 in UBC. MiR-31 was found to have suppressive effects on UBC cells, with ITGA5 identified as its direct target. Results from our *in vitro* and *in vivo* experiments indicated that miR-31 increased sensitivity of UBC to MMC through suppressing ITGA5 and downstream pathways. We also characterized the expression profile of miR-31 in UBC specimens, and analyzed its association with prognosis in the subset of patients with noninvasive UBC. Taken together, our results reveal the significant functions of miR-31 in UBC as well as the promising prospect of miR-31/ITGA5 axis as a novel therapeutic target.

## RESULTS

### MiR-31 is down-regulated in UBC tissues and its expression correlates with individual prognosis

**Figure 1:**
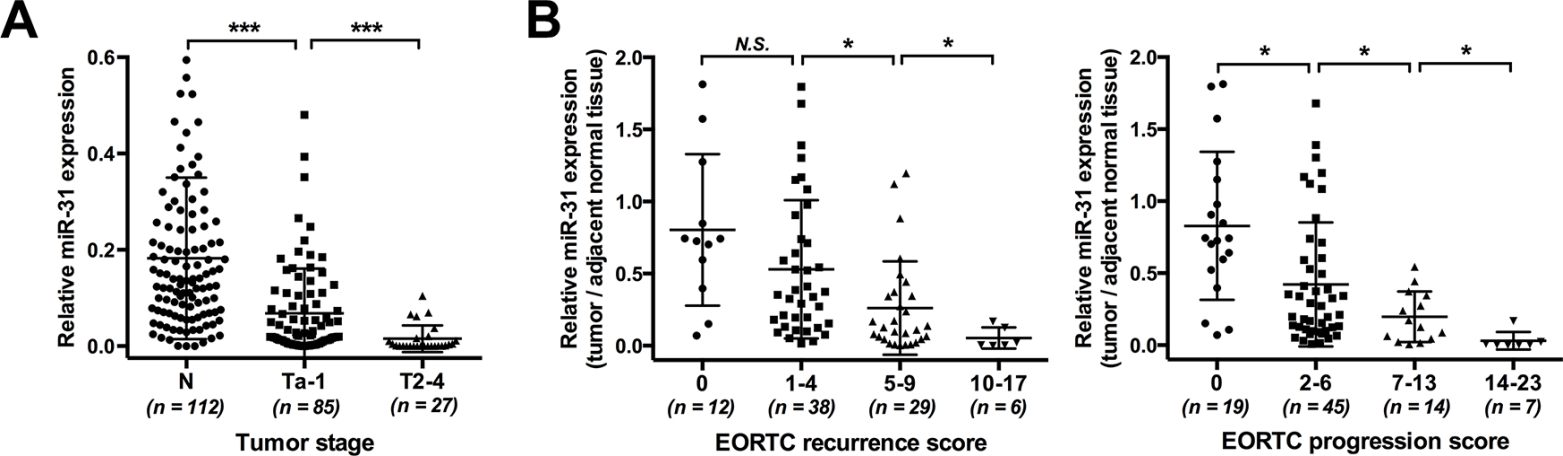
Down-regulation of miR-31 in UBC tissues and its association with risks of unfavorable prognosis (**A**) Detection of miR-31 expression was carried out by qRT-PCR assay in 112 pairs of UBC and normal urothelial samples; U6 snRNA served as an internal control. Expression levels of miR-31 were compared among urothelium (normal control, *n* = 112), noninvasive (stage Ta–1, *n* = 85) and invasive (stage T2–4, *n* = 27) tumor tissues. (**B**) The inverse correlation of recurrence (left panel) or progression (right panel) risk with miR-31 expression in noninvasive UBC. The relative expression of miR-31 in tumors was determined in comparison with that in adjacent normal urothelium; EORTC model was employed for risk stratification. Data are presented as mean ± SD. *N.S.*, *P* ≥ 0.05; *P < 0.05; ***P < 0.001.

To elucidate the role of miR-31 in UBC, quantitative real-time PCR (qRT-PCR) was first conducted to determine its expression in bladder urothelial tissues from specimens of 112 surgically treated patients. Compared with adjacent normal urothelium, significantly down-regulation of miR-31 was detected in UBC lesions. Moreover, invasive UBC displayed markedly lower expression levels than noninvasive phenotype (Figure 1A). We further evaluated the association of miR-31 with oncologic outcomes. The current study focused on cases with noninvasive UBC, which accounted for the majority (85/112) of our cohort. EORTC scoring model, a classical tool for prognostic prediction of noninvasive UBC, has been perfectly validated in Chinese patients receiving intravesical chemotherapy [21]. We divided the cohort into different prognostic risk groups based on model scores and found that the relative miR-31 expression was significantly inversely correlated with progression risk in noninvasive UBC. Similar relationship was observed regarding recurrence risk, except that there was no difference between low and intermediate-low risk groups (Figure 1B). These results suggest that down-regulation of miR-31 plays a role in UBC development and confers unfavorable prognosis, at least in noninvasive cases.

### Overexpression of miR-31 suppresses proliferation of UBC cells

**Figure 2:**
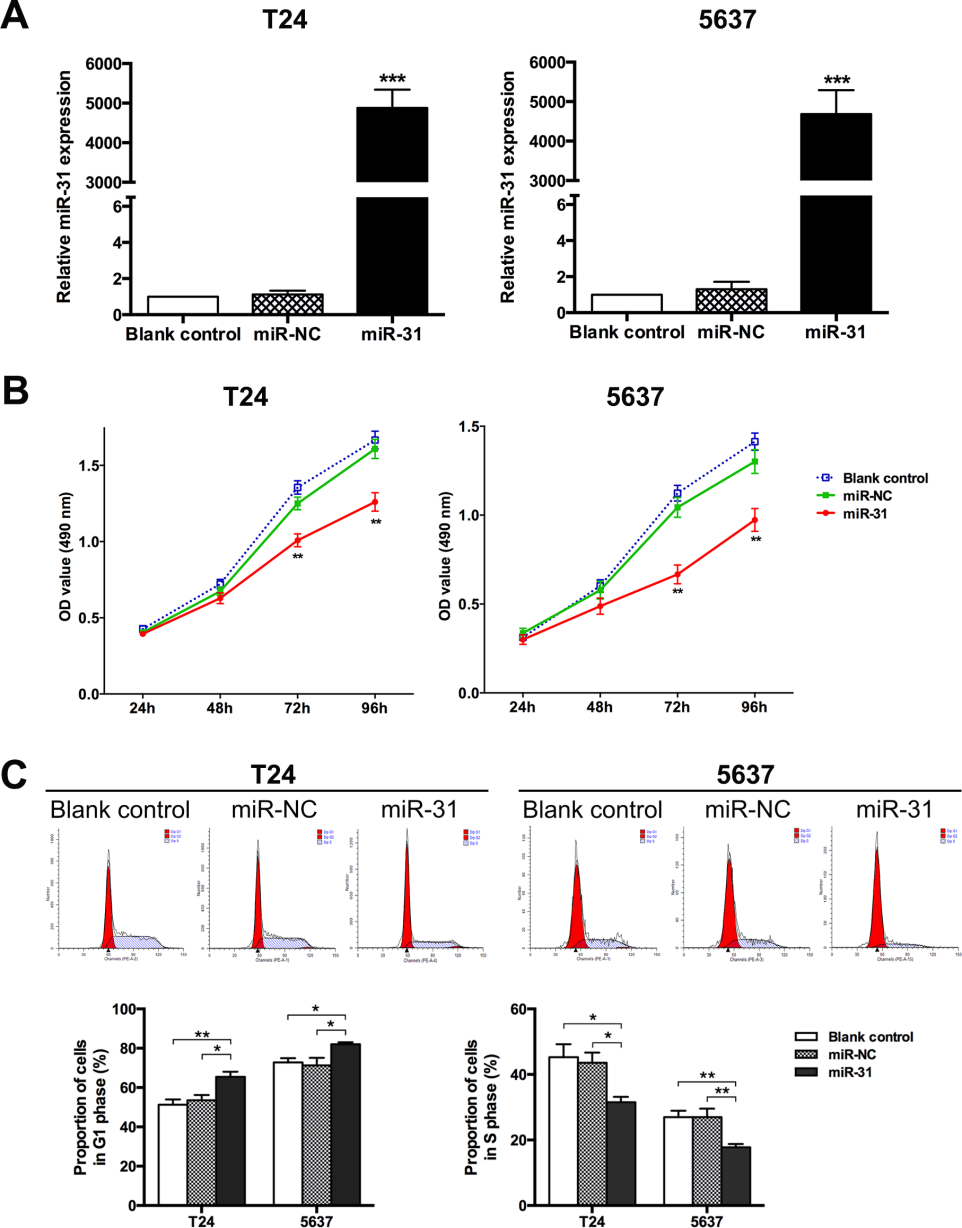
MiR-31 suppresses proliferation of UBC cells (**A**) T24 and 5637 cells were transfected with miR-31 mimic (50 nM); cells without transfection and those transfected with miR-NC served as blank and negative control, respectively. Expression of miR-145 was detected by qRT-PCR at 48 h after transfection; U6 snRNA served as an internal control. (**B**) At indicated time points after seeding, MTT assays were performed to evaluate the proliferation capacities of UBC cells for all groups. (**C**) Representative pictures of cell cycle distribution as detected by FCM analysis (upper panel) and comparison of the relative numbers of G1 phase cells (lower left panel) or S phase cells (lower right panel) among groups. Data are presented as mean ± SEM. *P < 0.05; **P < 0.01; ***P < 0.001.

Based on the aberrant expression of miR-31 in UBC tissues, researches on its functions were carried out. In T24 and 5637 cells, ectopic expression of miR-31 was induced by transfection with miRNA mimics and then verified by qRT-PCR (Figure 2A). As shown in Figure 2B, UBC cells without transfection and those transfected with scramble oligonucleotides (miR-NC) maintained healthy growth, whereas cells transfected with miR-31 mimics (miR-31) gradually lost viability and the numbers of viable cells were significantly reduced after 48 h of transfection. Cell cycle was also examined by flow cytometry (FCM) analysis, which showed that overexpression of miR-31 dramatically increased the percentage of G1 phase cells and decreased S phase cells (Figure 2C). Accordingly, miR-31 blocks G1 to S cell cycle transition and thereby suppresses UBC cell proliferation.

### Overexpression of miR-31 inhibits migration and invasion of UBC cells

**Figure 3:**
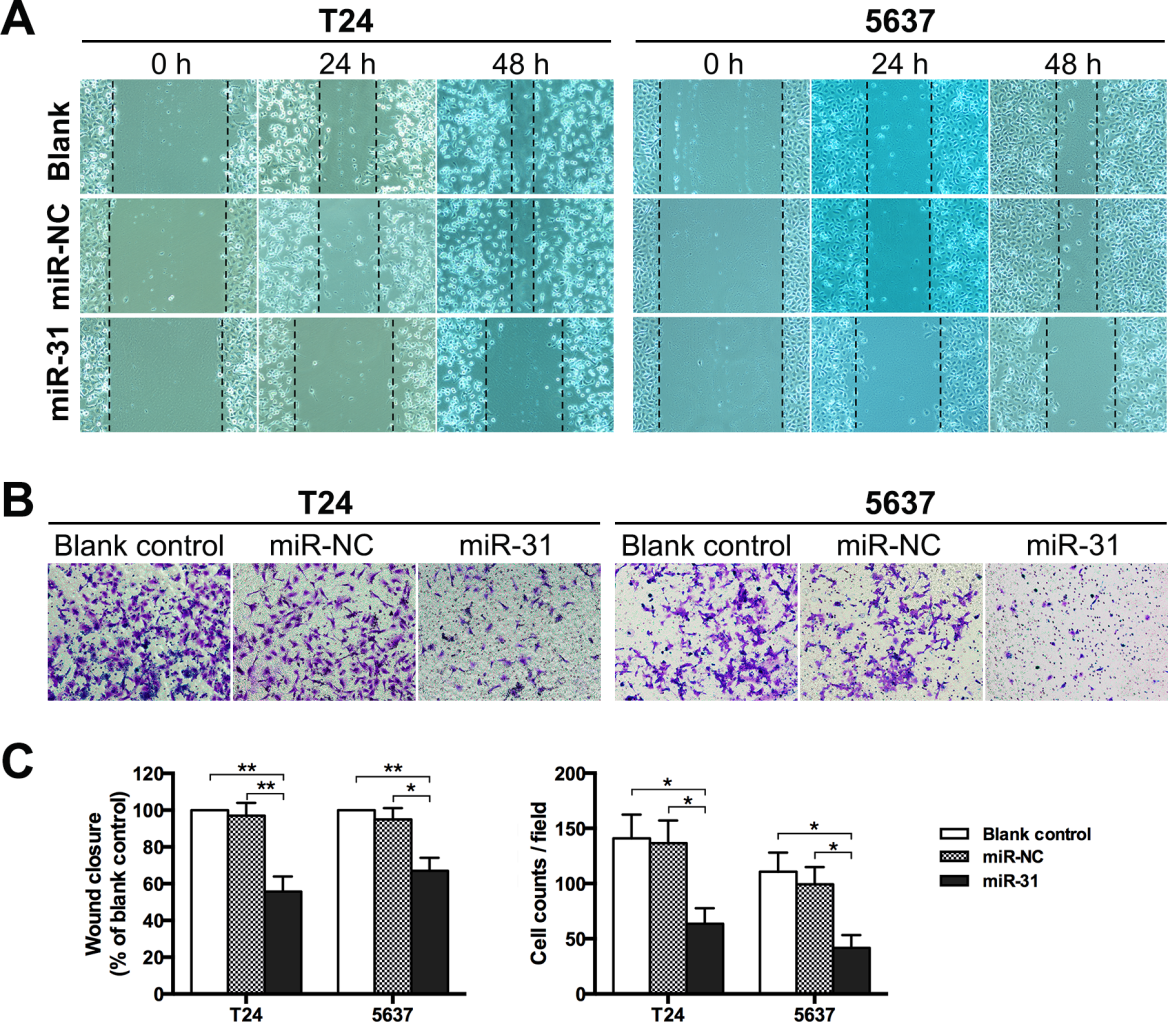
MiR-31 inhibits migration and invasion of UBC cells (**A**) T24 and 5637 cells were either without transfection (blank control) or transfected with miR-NC or miR-31, and then subjected to wound healing assay to determine the migration capacity at 24 h and 48 h following scarification, respectively. (**B**) Transwell assay was performed to evaluate the invasion capacity of UBC cells at 24 h after seeded onto the upper chamber. (**C**) Measurement of wound closure based on initial and residual (48 h) gap lengths in wound healing assay (left panel, relative to blank control group), and quantification of invaded cells in transwell assay (right panel); data are presented as mean ± SEM; *P < 0.05; **P < 0.01.

The effect of miR-31 expression on cell migration was evaluated by using wound healing assay. We observed that the wound closure of miR-31-expressing cells was significantly slower than that of other control groups (Figure 3A, 3C). Meanwhile, matrigel invasion assay was adopted to detect the ability of cell invasion, and there were significantly fewer cells that invaded through matrigel and migrated onto the lower surface of the membrane, in miR-31 transfectant (Figure 3B, 3C). These results indicate that miR-31 inhibits migration and invasion of UBC cells.

### MiR-31 directly suppresses ITGA5 expression in UBC cells

**Figure 4:**
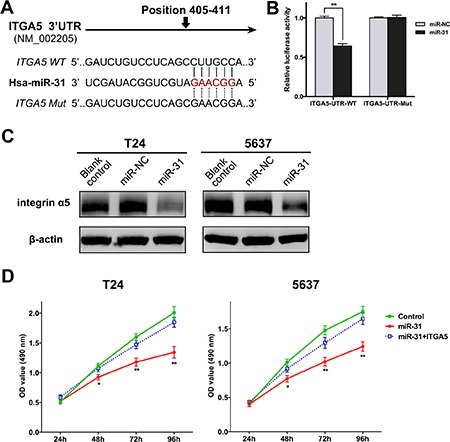
ITGA5 is a direct target of miR-31 in UBC cells (**A**) The potential miR-31 binding sites of wild-type ITGA5 3′UTR and the mutated sequences. (**B**) The relative luciferase activity was detected in T24 cells co-transfected with miR-31 (or miR-NC) and reporter plasmid carrying wild-type or mutant ITGA5 3′UTR. (**C**) Western blot assay was performed to determine the expression of ITGA5 protein in T24 and 5637 cells either without transfection (blank control), or transfected with miR-NC or miR-31. (**D**) MTT assay was performed to compare proliferation of UBC cells after co-transfection of miR-31 (or miR-NC) and ITGA5-expressing plasmid (or empty plasmid); cells co-transfected with miR-NC and empty plasmid served as negative control. Data are presented as mean ± SEM; *P < 0.05; **P < 0.01.

To identify the effector gene of miR-31, we performed bioinformatic analyses. As suggested by the TargetScan algorithm, ITGA5 mRNA has one theoretical miR-31 binding site within the 3′-untranslated region (3′UTR) (Figure 4A). Luciferase reporter assay was performed to confirm whether ITGA5 is a target of miR-31 in T24 cells. As shown in Figure 4B, co-transfection with miR-31 significantly reduced the luciferase activity of the reporter plasmid carrying wild-type ITGA5 3′UTR. In contrast, the suppressive effect was abolished when the miR-31 binding sequence in ITGA5 3′UTR was mutated. Furthermore, we validated above findings by examining ITGA5 expression at the protein level. After expression of miR-31, endogenous ITGA5 was remarkably down-regulated in both T24 and 5637 cells (Figure 4C).

According to above results, ITGA5 is a direct target of miR-31 in UBC cells. To verify whether miR-31 exerted inhibitory functions by targeting ITGA5, we performed rescue experiments by co-transfection of miR-31 with recombinant plasmid encoding ITGA5 (lacking 3′UTR) in UBC cells. MTT assay showed that cells expressing miR-31 alone proliferated at significantly slower rates. When miR-31 was co-expressed with ITGA5, cell proliferation was activated again (Figure 4D). It can be deduced that miR-31 inhibits proliferation of UBC cells by targeting ITGA5.

### MiR-31 overexpression increases sensitivity of UBC cells to MMC through suppressing ITGA5 and downstream signaling cascades

**Figure 5:**
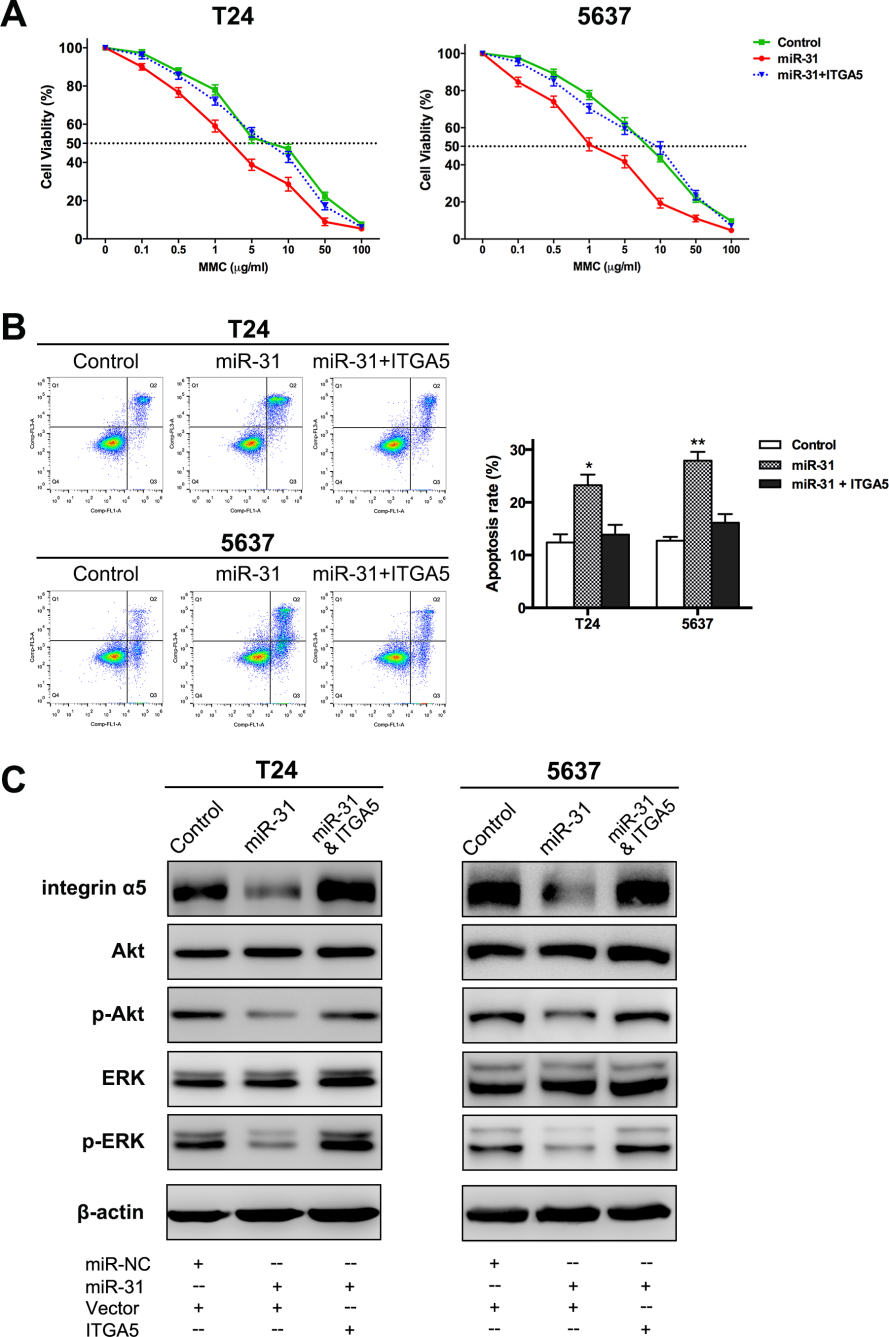
MiR-31/ITGA5 axis increases sensitivity of UBC cells to MMC via inactivating Akt and ERK pathways (**A**) Following 24 h treatment with MMC at different concentrations, MTT assay showed the survival rates of UBC cells co-transfected with miR-31 and empty plasmid were remarkably lower than those of cells co-transfected with miR-NC and empty plasmid (control); while co-transfection of miR-31 and ITGA5-expressing plasmid abrogated this effect. (**B**) Representative pictures of cell apoptosis distribution as detected by FCM analysis; region Q1, Q2, Q3 and Q4 shows dead, late apoptotic, early apoptotic and living cells, respectively; Q2 and Q3 are collectively regarded as apoptotic cells (left panel). Co-transfection of ITGA5-expressing plasmid basically eliminated the significant aggravation of MMC-induced apoptosis arising from miR-31 expression (right panel). (**C**) As indicated by western blot assay, overexpression of miR-31 significantly reduced the protein levels of ITGA5, phospho-Akt and phospho-ERK, while both Akt and ERK were reactivated again after ITGA5 expression was restored. Data are presented as mean ± SEM; *P < 0.05; **P < 0.01.

In previous study, we demonstrated that the engagement of integrin induced resistance to MMC in UBC cells adhering to fibronectin [18]. Herein, after identifying ITGA5 as a target gene of miR-31, we next sought to determine whether miR-31 modulated the sensitivity to MMC. To this aim, MTT assay and FCM analysis were employed to detect MMC-induced cytotoxicity and apoptosis, respectively. After exposure to MMC at different concentrations, miR-31-expressing UBC cells showed significantly lower survival rates and higher IC_50_ values of MMC, compared with miR-NC-transfected cells (Figure 5A). FCM analysis demonstrated that miR-31 overexpression could promote MMC-induced apoptosis in both T24 and 5637 cells (Figure 5B). Simultaneously, another group of UBC cells was co-transfected with miR-31 and ITGA5-expressing plasmid. The effects of chemosesitization caused by miR-31 were markedly reversed after restoring ITGA5 expression, as indicated by the survival and apoptotic rates approximate to baseline levels (Figure 5A–5B). Thus, miR-31 exerts these functions through suppressing ITGA5.

Western blot assays of intracellular signaling cascades were also performed to confirm the involvement of miR-31/ITGA5 axis in MMC chemosensitization and to address the underlying mechanisms. Following MMC treatment, overexpression of miR-31 resulted in suppression of phosphorylation of Akt and ERK, possibly secondary to the down-regulation of ITGA5. When ITGA5 expression was restored, both Akt and ERK were activated again (Figure 5C). Collectively, miR-31 increases sensitivity to MMC by suppressing ITGA5 and inactivating Akt and ERK pathways in UBC cells.

### MiR-31 overexpression attenuates tumor growth and increases sensitivity to MMC *in vivo*

**Figure 6:**
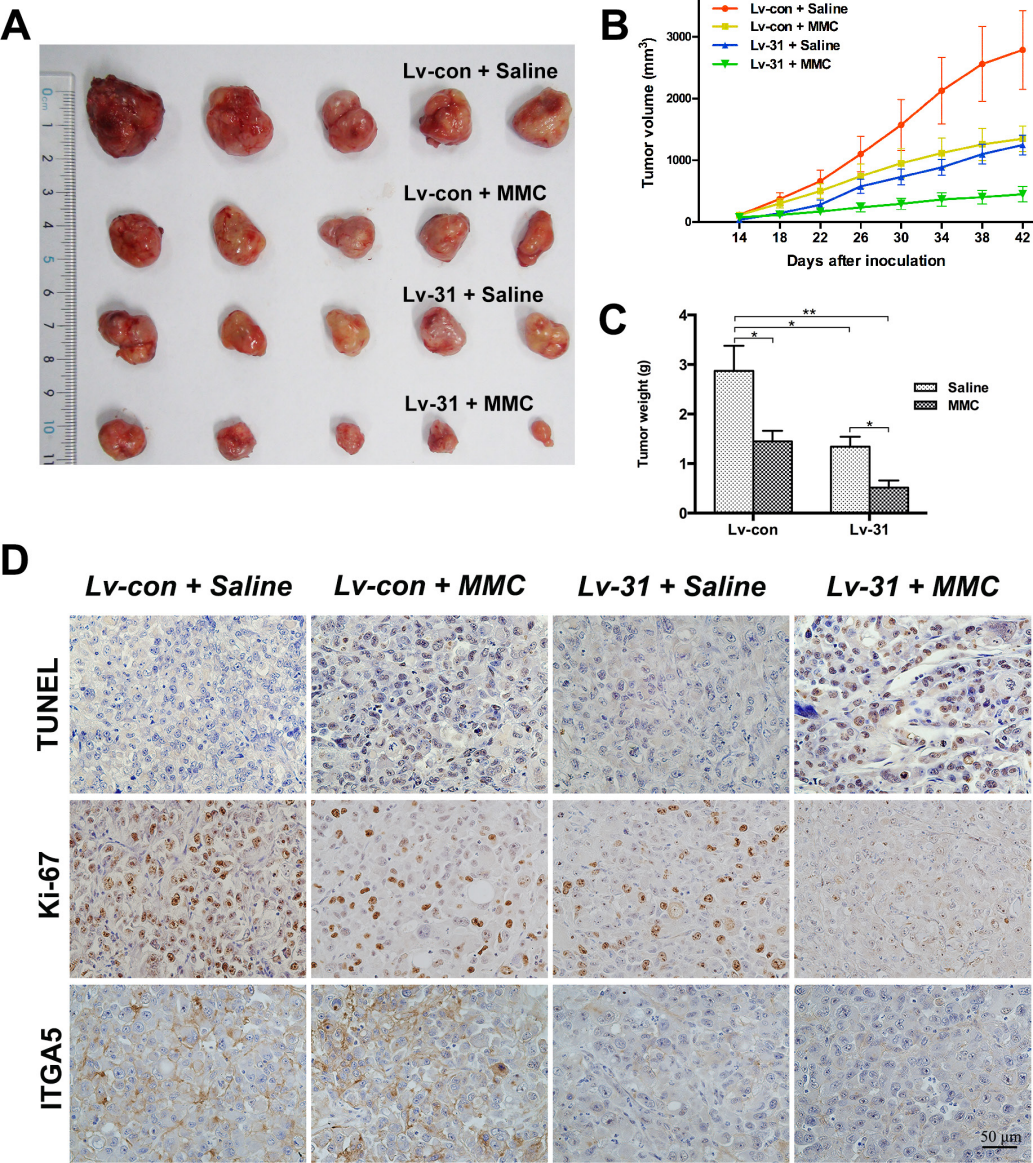
MiR-31 attenuates xenograft tumor growth and exhibits a synergistic effect with MMC treatment *in vivo* (**A**) Representative photographs of dissected xenograft tumors from nude mice after sacrificed. (**B** and **C**) Graphics representing tumor volumes at indicated days after inoculation and tumor weights at the end of the experiment; data are presented as mean ± SEM; *P < 0.05; **P < 0.01. (**D**) Representative images from multiple fields of xenograft tumor sections with TUNEL, Ki-67 or ITGA5 staining; bars: 50 μm.

Given that miR-31 acted as an UBC suppressor *in vitro*, we further assessed its effect *in vivo*. By using a lentiviral system, T24 cells with stable overexpression of miR-31 were established and then subcutaneously inoculated into nude mice to induce tumor xenografts. As shown in Figure 6A–6C, tumors with miR-31-expressing cells (designated as miR-31 tumors) grew at a slower rate. Significant reductions in tumor size and weight were observed at the termination of experiment, as compared with tumors with empty-vector-expressing cells (designated as vector tumors). MMC treatment combined with miR-31 overexpression provided the strongest suppressive effect on tumorigenicity, and the tumor-inhibitory rate of MMC was higher in miR-31 tumors than in vector tumors (61.7% vs. 49.5%). Pathologic analyses were also performed in tumor specimens (Figure 6D). TUNEL assays showed that, although the percentages of apoptotic cells were similar between miR-31 and vector tumors, MMC treatment resulted in dramatically more apoptosis in miR-31 tumors. Ki67 staining showed markedly fewer proliferative cells in miR-31 tumors and MMC-treated vector tumors, with the fewest in MMC-treated miR-31 tumors. Meanwhile, miR-31 tumors displayed lower expressions of ITGA5 than vector tumors, whether or not MMC was administered. Thus, miR-31 inhibits formation of UBC xenografts and improves therapeutic effects of MMC, and these results support our *in vitro* findings.

## DISCUSSION

There has been increasing amount of knowledge regarding some key roles of miRNAs in cancer biology. In this study, specific functions and mechanisms of miR-31 in UBC were determined for the first time. We demonstrated that miR-31 served as an UBC suppressor through negative regulation of cell cycle, migration and invasion. The member of integrin family, ITGA5, was identified as a direct target of miR-31 in UBC cells. Moreover, activation of miR-31/ITGA5 axis increased sensitivity of UBC to MMC both *in vitro* and *in vivo*. Our results provide strong evidences for exploring miR-31 as a novel strategy in UBC treatment.

The expression of miR-31 is altered among different cancers. In colorectal and lung cancers, miR-31 is highly expressed, whereas down-regulation has also been found in other malignancies [7]. The miR-31 gene is located at chromosome 9p21, a region where homozygous deletion is commonly detected in UBC [22, 23]. Consistently, microarray data suggest that miR-31 homozygous loss frequently occurs in UBC tissues [24]. In our study, UBC lesions were characterized by the reduced miR-31 expression. Particularly, the invasive phenotype showed more significant down-regulation. Our data successfully confirm two separate previous reports that conducted comparative studies of tumor vs. non-tumor and invasive vs. noninvasive samples, respectively [14, 15]. The differential expression suggests a role for miR-31 in both early and late stages of UBC development. MiR-31 dysregulation may affect oncologic outcomes of several malignancies, including glioblastoma, cervical, colorectal and liver cancer [8, 25–27]. Segersten et al. [28] reported that the decreased expression of miR-31 correlated with pathologic progression of Ta UBC. By using the classical EORTC prognostic model, our results suggest that lower miR-31 expression levels tightly correlate with higher risks of recurrence and progression in noninvasive UBC. Hence, this miRNA could be a prognostic marker for patients with UBC.

In the present study, miR-31 overexpression elicited inhibition of proliferation of UBC cells, and the suppressive effects were validated in xenografts. Similar phenomena were observed in medulloblastoma, prostate and liver cancers [8–10]. We also found that miR-31 disrupted the migratory and invasive processes in UBC cells, just as in breast, esophageal and gastric cancers [11–13]. This is consistent with the fact that invasive UBC tissues contain significantly lower levels of miR-31. MiRNAs function via specific binding to complementary site within the 3′UTR of target genes. Here, luciferase assay indicated that miR-31 targeted ITGA5 directly in T24 cells. This finding was further confirmed by immunoblot assays and ITGA5 staining of xneografts. Moreover, by rescue experiment, miR-31/ITGA5 axis was demonstrated to be responsible for miR-31-induced inhibition of proliferation in UBC cells. ITGA5 exhibits either tumor-promotional or inhibitory properties in different cancers [29]. For UBC, higher expression of ITGA5 is detected in more malignant tumors [30]. In T24 cells, ITGA5 overexpression facilitates cellular invasion [31]. In other UBC cell lines (UMUC3, Lul2), ITGA5 controls focal adhesion kinase/Akt pathway, a key cascade of cell survival, proliferation, migration and invasion [32, 33]. All these suggest that ITGA5 acts as oncogene in UBC. Although one miRNA regulate multiple genes, the inhibitory functions of miR-31 in UBC should be attributed, in part at least, to the suppression of ITGA5.

Previous studies have focused on influences of miRNAs on chemosensitivity in UBC. For example, miR-34a can sensitize UBC cells to cisplatin by suppressing Cdk6 and sirtuin-1 [34]. Cisplatin also up-regulates miR-34a via promoter demethylation, and miR-34a suppresses CD44 expression and in turn improves chemotherapeutic effect [35]. In contrast, miR-193a-3p confers multi-chemoresistance by affecting several pathways, like DNA damage response and oxidative stress [36–40]. Our study enriches this library by finding miR-31 increasing the sensitivity of UBC to MMC, another chemotherapeutic agent. Cellular interaction with ECM could generate chemoresistance, namely cell adhesion mediated drug resistance (CAM-DR) [41]. In UBC cells, adhesion to fibronectin induces MMC resistance, and targeting ITGB1 abrogates the adhesion and increase drug sensitivity [18, 42]. The present study demonstrates that miR-31 can affect this process. Apparently, miR-31 expression reverses CAM-DR by down-regulating ITGA5, the important subunit of fibronectin receptor (integrin α5β1). Further analysis showed that miR-31 might sensitize UBC cells to MMC by inactivating survival-related Akt and proliferation-related ERK pathways. Our results echo another study that ITGA5 simultaneously controlled these two critical signaling cascades in epidermoid carcinoma cells [43]. In recent years, integrin antagonists, like the anti-ITGA5 antibody volociximab, have become promising agents either as single drug or in combination with chemotherapy [44–46]. By suppressing ITGA5, up-regulated miR-31 represents a novel therapeutic approach for UBC. It was reported that miR-31 inhibits expressions of fibronectin and other ECM proteins [8]. Such changes would also help abrogate the CAM-DR and confer favorable response to chemotherapy.

In conclusion, our findings expatiate functional mechanisms and clinical significance of miR-31 as a tumor suppressor in UBC. Specifically, miR-31 expression brings about enhanced sensitivity of UBC to MMC by suppressing ITGA5 and downstream pathways. These data suggest that miR-31 may serve as both prognostic biomarker and novel therapeutic target for improved UBC management.

## MATERIALS AND METHODS

### Patients and tissue samples

All procedures of this study were reviewed and approved by institutional ethics committee. Paired samples of tumor/non-tumor urothelial tissues were obtained from 112 patients who underwent TURBT, partial or radical cystectomy at our center, between June 2014 and June 2015. Samples were immediately snapped frozen in liquid nitrogen and stored at −80°C until RNA extraction. Clinicopathologic data were collected for all patients, among whom 85 had noninvasive UBC and recieved TURBT or partial cystectomy followed by intravesical instillation of chemotherapy. To perform prognostic analyses, individual recurrence or progression risk score was calculated by using the prognostic model from European Association of Urology (EAU) guidelines [20], and noninvasive UBC patients were hereby stratified into different risk groups.

### Cell culture and miRNA transfection

Human UBC cell lines (T24 and 5637), obtained from Type Culture Collection of the Chinese Academy of Science (Shanghai, China), were cultured in RPMI 1640 medium containing 10% fetal bovine serum (FBS, Gibco, Carlsbad, CA, USA), 100 units of penicillin/mL and 100 μg of streptomycin/mL (complete medium). Cells were incubated in a humidified atmosphere of 5% CO_2_ and 95% air at 37°C. MiR-31 mimic and scramble oligonucleotides, termed as miR-31 and miR-NC respectively, were both synthesized by RiboBio biotechnology (Guangzhou, China). MiRNA transfection was carried out using lipofectamine 2000 reagent (Invitrogen, Carlsbad, CA, USA), following the manufacturer's instruction.

### RNA extraction and quantitative real-time PCR (qRT-PCR)

Total RNA was extracted from tissues or UBC cells by using Trizol reagent (Invitrogen). Specific stem-loop reverse transcription primers were used for cDNA synthesis of miR-31 and U6 snRNA with PrimeScript II 1st Strand cDNA Synthesis Kit (TaKaRa, Tokyo, Japan). Real-time PCR was performed using SYBR Premix Ex Taq II Kit (TaKaRa), on an Applied Biosystems 7500 Real-time PCR system (Applied Biosystems, Carlsbad, CA, USA). The Bulge-loop primers and qRT-PCR primers for miR-31 and U6 snRNA were synthesized and purchased from RiboBio. The expression of miR-31 was normalized by U6 snRNA.

### Cell proliferation assay

Cell viability was evaluated by measuring the absorbance of 3-(4, 5-dimethylthiazol-2-yl)-2, 5-diphenyltetrazolium bromide (MTT) (Invitrogen). About 2 × 10^3^ UBC cells were plated in each well of a 96-well plate and transfection was performed after overnight incubation. At indicated time points after seeding, the numbers of viable cells were evaluated by MTT assay according to the manufacturer's protocol. The absorbance values of experimental wells were read at 490 nm on a spectrophotometer and normalized by subtracting the mean of blank well values. These experiments were performed in sextuplicate.

### Cell cycle analysis

The cell cycle analysis was carried out by flow cytometry (FCM). UBC cells were seeded onto a 6-well plate and then transfected with miR-31 or miR-NC. At 48 h after transfection, cells were collected and fixed with 70% ice ethanol overnight at 4°C. The centrifuged cells were subsequently stained with propidium iodide/RNase buffer (BD Biosciences, San Jose, CA, USA), according to the manufacturer's instruction. The data were collected and analyzed on a FACScalibur flow cytometer with the CellQuest software (BD Biosciences). These experiments were performed a minimum of three times.

### Cell migration assay

The effect of miR-31 on cell migration was investigated by wound healing assay. Briefly, transfection with miR-31 or miR-NC was conducted in UBC cells on a 6-well plate. Upon reaching confluence, the cell monolayer was scraped straightly by using a micropipette tip and washed with phosphate-buffered saline (PBS) to remove cell debris. The scraped monolayer was incubated in serum-free medium for 48 h, and gap distances at indicated time points after wounding were measured under a light microscope.

### Cell invasion assay

The cell invasion activity was measured by using 24-well transwell chamber with 8-μm-pore insert coated with matrigel (Corning, NY, USA). Approximately 5 × 10^4^ UBC cells, which were resuspended in serum-free medium after 48 h of transfection, were plated in the upper compartment of chamber. Complete medium as the source of chemo-attractants was added into the lower chamber. After incubated for 24 h, cells remaining on the upper side of inserts were gently scraped off and invaded cells on the lower surface were fixed in 4% paraformaldehyde and stained with 0.1% crystal violet. For each insert, three random fields were selected, and the numbers of invaded cells were counted under the microscope.

### Luciferase reporter assay

The human ITGA5 3′UTR containing the putative miR-31 binding sites was amplified by PCR with the forward primer, 5′-CCGCTCGAGGAAACAAACTTGG AAAGATAACT-3′, and the reverse primer, 5′-AT AAGAATGCGGCCGCCCCATTTGAGTTCTGATTC-3′. The mutant ITGA5 3′UTR with point substitutions in the miR-31 binding sites was synthesized by Invitrogen. The product was cloned into the Xho I and Not I sites of a psi-CHECK2 luciferase reporter vector (Promega, Madison, WI, USA). All constructs were sequence verified.

Then T24 cells were plated in 96-well plates and transfected with wild-type or mutant ITGA5-3′UTR vector (100 ng per well), along with miR-31 or miR-NC (30 pmol per well). Cells were harvested and lysed for luciferase assay after 48 h of transfection. The activities of *Renilla* and firefly luciferases were determined by using the Dual-Luciferase Reporter Assay System (Promega). Values were normalized with firefly luciferase activity.

### Construct for ITGA5 overexpression

The ITGA5 coding sequence was amplified by PCR using the forward primer, 5′-CTAGTCTAGAATGGGGAGC CGGACGCC-3′, and thereverse primer, 5′-ATAAGAAT GCGGCCGCTCAGGCATCAGAGGTGGCTGG-3′. To generate ITGA5-expressing plasmid, the PCR-amplified fragment was cloned into the pLVX-IRES-Puro vector (Clontech, Mountain View, CA, USA) using Xba I/Not I digestion. The construct product was validated by sequencing. Transfection of plasmids was carried out using lipofectamine 2000.

### *In vitro* MMC sensitivity assay

UBC cells were seeded onto a 6-well plate and co-transfected with miR-31 (or miR-NC) and ITGA5-expressing plasmid (or empty plasmid). After 48 h of transfection, viable cells were transferred to another 6-well plate pre-coated with human plasma fibronectin (Gibco) and then treated with 100 μg/mL of MMC (Sigma, St. Louis, MO, USA) for 2 h, as previously reported [18]. After another 24 h of culture in complete medium without MMC, UBC cells were harvested for apoptosis assessment. Double staining of fluorescein isothiocyanate (FITC) labeled annexin-V and propidium iodide was performed by using Annexin V-FITC Apoptosis Detection kit (Dojindo, Kunamoto, Japan), according to the manufacturer's protocol. The stained cell suspension was then subjected to FCM analysis, and UBC cells were discriminated into living, dead, early apoptotic and late apoptotic cells, with distribution determined by the FlowJo software (Treestar, Ashland, OR, USA). These experiments were performed three times.

Besides, MTT assay was employed to evaluate the influence of MMC on cell viability. Briefly, UBC cells after transfection were transferred to a fibronectin-coated 96-well plate, with 0.1–100 μg/mL MMC added in culture for 24 h. The cell survival rate was calculated as a percentage of the corresponding control (0 μg/mL MMC) absorbance. These experiments were performed in sextuplicate.

### Protein extraction and western blot assay

Cells after transfection or MMC treatment were washed in cold PBS twice and then lysed on ice with RIPA buffer containing protease and phosphatase inhibitors (Thermo Fisher Scientific, Franklin, MA, USA). Protein concentrations were measured by using BCA Protein Assay Kit (Beyotime, China). Twenty micrograms of denatured protein were loaded on SDS-PAGE gel and transferred onto polyvinylidene difluoride membranes (Millipore, Billerica, MA, USA). Then the membranes were blocked for 2 h in Tris buffered saline with 0.1% tween (TBST) containing 5% bovine serum albumin (Sigma), and subsequently incubated overnight at 4°C with primary antibodies against ITGA5, Akt, phospho-Akt, ERK, phospho-ERK or β-actin (all from Cell Signaling Technology, Beverly, USA) at recommended dilutions. After complete washing in TBST, membranes were incubated with the corresponding horseradish peroxidase-conjugated secondary antibodies at room temperature for 1 h and visualized with ECL substrates (Millipore). β-actin was used as a loading control.

### Lentiviral-mediated miR-31 overexpression and xenograft model

Lentiviral vector pLenti6.3/V5-GW/EmGFP (Invitrogen) encoding hsa-miR-31 precursor was constructed, and verification, production and purification of lentivirus were performed in Novobio biotechnology (Shanghai, China). The packaged lentivirus was named lv-31, and those generated from the empty (not carrying target gene) vector served as control (designated as lv-con). To perform transduction, T24 cells were seeded onto a 6-well plate, cultured to achieve 50–70% confluence and then infected with 5 × 10^6^ transducing units of lv-31 or lv-con at the presence of polybrene (Sigma). The medium was changed at 24 h and the infection efficiency was measured under a fluorescent microscope at 72 h. Stable cells with or without miR-31 overexpression were selected and maintained in medium containing 5 μg/mL blasticidin S (Sigma).

Animal experiments were performed according to the protocol approved by the insititutional animal experimentation committee. Twenty 4-week-old female BALB/c nude mice were randomized into four equal groups. Mice of two groups were each subcutaneously inoculated with 2 × 10^6^ T24 cells expressing miR-31, and others with equivalent number of cells expressing empty vector. The size of tumors and body weight of mice were measured every other day, and tumor volume was calculated with the formula length × width^2^ × 0.5. Intraperitoneal administration of MMC (3 mg/kg) or physiological saline started when tumor became palpable. At the end of experiments, all mice were sacrificed and tumors were excised and weighed. Tumor tissues were obtained for further analyses. Tumor-inhibitory rate of MMC (with or without miR-31 overexpression) was calculated with the formula (1 − average tumor weight of MMC-treated mice/average tumor weight of saline-treated mice) × 100%.

### TUNEL and immunohistochemical staining

Paraffin-embedded xenograft tumors were made into 5-μm sections and prepared for staining. Terminal deoxynucleotidyl transferase mediated dUTP nick-end labeling (TUNEL) assay was performed using DeadEnd™ Colorimetric TUNEL System (Promega), according to the manufacturer's instruction. The primary antibodies against Ki-67 (Santa Cruz Biotechnology, Santa Cruz, CA, USA) or ITGA5 at recommended dilution were added to the slides, which were thereafter incubated overnight at 4°C. Normal IgG instead of primary antibodies was as negative control. Then the slides were washed with PBS and incubated for 2 h at room temperature by using Envision kit (DAKO, Carpinteria, CA, USA). For each index, two independent observers made an assessment of sections based on the number and intensity of cell staining, as described previously [47].

### Statistical analysis

All statistical analyses were performed by SPSS 16.0 software (SPSS Inc., Chicago, IL, USA). The one-way ANOVA and the χ2 test were employed to compare quantitative data and categorical data, respectively. In all tests, *P* < 0.05 was considered statistically significant.
